# Enhanced Photovoltaic Performance of Dye-Sensitized Solar Cells by Efficient Near-Infrared Sunlight Harvesting using Upconverting Y_2_O_3_:Er^3+^/Yb^3+^ Phosphor Nanoparticles

**DOI:** 10.1186/s11671-015-1030-0

**Published:** 2015-08-12

**Authors:** Peng Du, Joo Ho Lim, Jung Woo Leem, Sung Min Cha, Jae Su Yu

**Affiliations:** Department of Electronics and Radio Engineering, Kyung Hee University, Yongin, 446-701 Republic of Korea

**Keywords:** Upconversion, Rare-earth, Luminescence, Dye-sensitized solar cells

## Abstract

We report the efficiency enhancement in dye-sensitized solar cells (DSSCs) using Er^3+^/Yb^3+^-co-doped Y_2_O_3_ (i.e., Y_2_O_3_:Er^3+^/Yb^3+^) phosphor nanoparticles, prepared by a simple and cost-effective urea-based homogeneous precipitation method, for efficient near-infrared (NIR) sunlight harvesting. Under the light excitation at a wavelength of 980 nm, the as-prepared samples exhibited strong upconversion emissions at green and red visible wavelengths. To investigate the influence of Y_2_O_3_:Er^3+^/Yb^3+^ nanoparticles on the photovoltaic performance of DSSCs, the phosphor nanoparticles were incorporated into titanium dioxide films to form a composite photoelectrode. For the resulting DSSCs, the increased power conversion efficiency (*PCE*) of 6.68 % was obtained mainly by the increased photocurrent of *J*_SC_ = 13.68 mA/cm^2^ due to the light harvesting enhancement via the NIR-to-visible upconversion process (cf., *PCE* = 5.94 %, *J*_SC_ = 12.74 mA/cm^2^ for the reference DSSCs without phosphor nanoparticles), thus, indicating the *PCE* increment ratio of ~12.4 %.

## Background

Dye-sensitized solar cells (DSSCs), which can convert solar energy into electric energy by the photovoltaic effect, have been intensively studied as a promising candidate for the next-generation photovoltaic devices because of their simple structure, good stability, low manufacturing cost, and eco-friendly feature [1–5]. Over the past few years, although significant progress in DSSCs has been achieved, the power conversion efficiency (*PCE*) is still not satisfied compared to the silicon-based solar cells, which limits their further applications [6–8]. As is well known, the *PCE* of the DSSCs is strongly dependent on the light absorption ability of dyes such as N3, N-719, N-749, etc., which usually absorb energy from the relatively narrow sunlight spectrum in the visible wavelength range of 400–800 nm [3, 8]. Therefore, if the near-infrared (NIR) light (>800 nm) which comprises nearly 50 % in the sunlight could be converted into the visible light and reabsorbed by dyes, the *PCE* of DSSCs would be further improved.

Up to date, enormous methods have been carried out to improve the performances of solar cells, by incorporating with nanowire particles and upconverting materials [9–11]. In particular, the introduction of upconverting nanoparticles into DSSCs devices was considered as an alternative method to improve the efficiency of DSSCs. Since they can convert the low energy photons (NIR light) into the high energy ones (visible light), the enhanced solar energy generation of DSSCs can be obtained due to the increased visible light absorption in dyes [12–14]. Demopoulos et al. reported that the *PCE* enhancement by 10 % was achieved using β-NaYF_4_:Er^3+^/Yb^3+^ nanoplatelets as the upconverting layer in DSSCs [13]. In addition, Wu et al. also showed the potential application of the Er^3+^/Yb^3+^-co-doped TiO_2_ upconverting nanoparticles in DSSC, exhibiting a boosted *PCE* of 7.05 % (i.e., *PCE* = 6.41 % for the pristine DSSC) [15]. Nevertheless, these obtained results are still far away from the practical application, so more efforts are required.

Recently, rare-earth (RE) ions doped nanomaterials were intensively investigated, and it was revealed that the luminescent properties of these RE ions doped nanomaterials can be modified by adjusting the size, shape, and phase of particles [16–18]. Among these nanomaterials, yttrium trioxide (Y_2_O_3_) is widely used as the optical host material owing to its high melting point, high thermal stability, and low toxicity [19, 20]. Furthermore, according to the Raman spectra, the Y_2_O_3_ has low phonon energy as low as ~600 cm^−1^ [21], which results in the high probability of the radiative transition. From this, it is expected that strong upconversion (UC) emissions could be obtained in RE ions doped Y_2_O_3_ material system. On the other hand, Er^3+^ ions, as a member of trivalent RE ions, have drawn considerable attention due to their unique green and red emissions corresponding to (^2^H_11/2_, ^4^S_3/2_) → ^4^I_15/2_ and ^4^F_9/2_ → ^4^I_15/2_ transitions, respectively. Moreover, Yb^3+^ ions are usually co-doped with Er^3+^ ions, as a sensitizer, to improve the luminescent properties because of their strong absorption in the NIR wavelength region and efficient energy transfer (ET) from the Yb^3+^ to Er^3+^ ions [22]. Also, the light scattering properties can be affected by the nanoparticles with the size ranging from 200–1000 nm [2, 13]. In this work, the upconverting Er^3+^/Yb^3+^-co-doped Y_2_O_3_ (abbreviated as Y_2_O_3_:Er^3+^/Yb^3+^) nanoparticles were prepared by a urea-based homogeneous precipitation method and their structural and optical properties were investigated. After incorporating the Y_2_O_3_:Er^3+^/Yb^3+^ into TiO_2_ nanocrystalline films to form a composite photoelectrode in DSSCs, for the fabricated devices, the current density-voltage (*J*-*V*) characteristics and the incident photon to current conversion efficiency (*IPCE*) spectra were explored.

## Methods

### Sample Preparation

To obtain the optimum UC emission property [23], both the Er^3+^ and Yb^3+^ ion concentrations were fixed at 1 mol%, and the Y_2_O_3_:Er^3+^/Yb^3+^ nanoparticles were successfully synthesized via a facile and simple urea-based homogeneous precipitation method, followed by appropriate thermal treatment. Briefly, stoichiometric amounts of yttrium nitrate hexahydrate (Y(NO_3_)_3_ · 6H_2_O, 99.8 %), erbium nitrate pentahydrate (Er(NO_3_)_3_ · 5H_2_O, 99.9 %), and ytterbium nitrate pentahydrate (Yb(NO_3_)_3_ · 5H_2_O, 99.9 %) were weighted and dissolved in 200 ml of deionized (DI) water to form a transparent solution. After that, moderate urea was added, and the mixed solution was sealed in a beaker, and then it was heated at 80 °C for 3 h under vigorous mechanical stirring. Subsequently, the precursor was centrifuged and washed with DI water and alcohol for several times to remove the remained ions. Finally, the precipitate was sintered at 800 °C for 3 h, thus, yielding the Y_2_O_3_:Er^3+^/Yb^3+^ nanoparticles. For the fabrication of DSSCs, the cleaned fluorine doped tin oxide (FTO)-deposited glass substrates were used. Firstly, TiO_2_ colloids (PST-18NR) were coated on the FTO surface to form a TiO_2_ film with a thickness of ~5 μm by a doctor-blade method, and then the samples were sintered at 500 °C for 2 h. The TiO_2_ colloids (PST-400C) mixed with 1 wt% Y_2_O_3_:Er^3+^/Yb^3+^ nanoparticles were subsequently screen-printed on the TiO_2_ film, which creates a 5-μm-thick TiO_2_ + Y_2_O_3_:Er^3+^/Yb^3+^ layer. Afterwards, the as-prepared film was soaked in the N-719 dye solution (3 × 10^−4^M in ethanol) for 24 h. For comparison, a dye-sensitized TiO_2_ film without Y_2_O_3_:Er^3+^/Yb^3+^ nanoparticles was also prepared. Meanwhile, the platinum (Pt) counter electrode was prepared on the FTO glass using Pt paste (Dyesol, counter PT-1), followed by heating at 500 °C for 2 h. Lastly, the DSSC devices were assembled by an injection of electrolyte (Dyesol, electrolyte HPE) and a sealing process with the help of a hot press.

### Characterization

The phase structure of the fabricated nanophosphor samples was analyzed by using an X-ray diffractometer (XRD; Mac Science, M18XHF-SRA) with Cu Kα (*λ* = 1.5402 Å) radiation, and the JADE software was applied to analyze the XRD data. The structural morphology was observed by using a transmission electron microscope (TEM; JEM-2100 F, JEOL). The room-temperature UC spectrum was checked by using a fluorescence spectrophotometer (Ocean optics USB 4000) under the excitation of a laser light at a wavelength (*λ*) of 980 nm with a pump power of 660 mW. The optical transmission and reflection spectra of dye-sensitized photoanodes with and without Y_2_O_3_:Er^3+^/Yb^3+^ phosphor nanoparticles were characterized by using a UV–vis-NIR spectrophotometer (Cary 5000, Varian). The J-V curves were measured by using a photocurrent system consisting of a solar simulator (ABET, SUN 3000) with a 1000 W Xe short-arc lamp and a source meter (Keithley 2400). The *IPCE* spectra from 300 to 800 nm were evaluated by using a 300 W xenon arc lamp as the light source coupled to a monochromator (TLS-300× xenon light source, Newport) with an optical power meter (2935-c, Newport).

## Results and Discussion

**Fig. 1:**
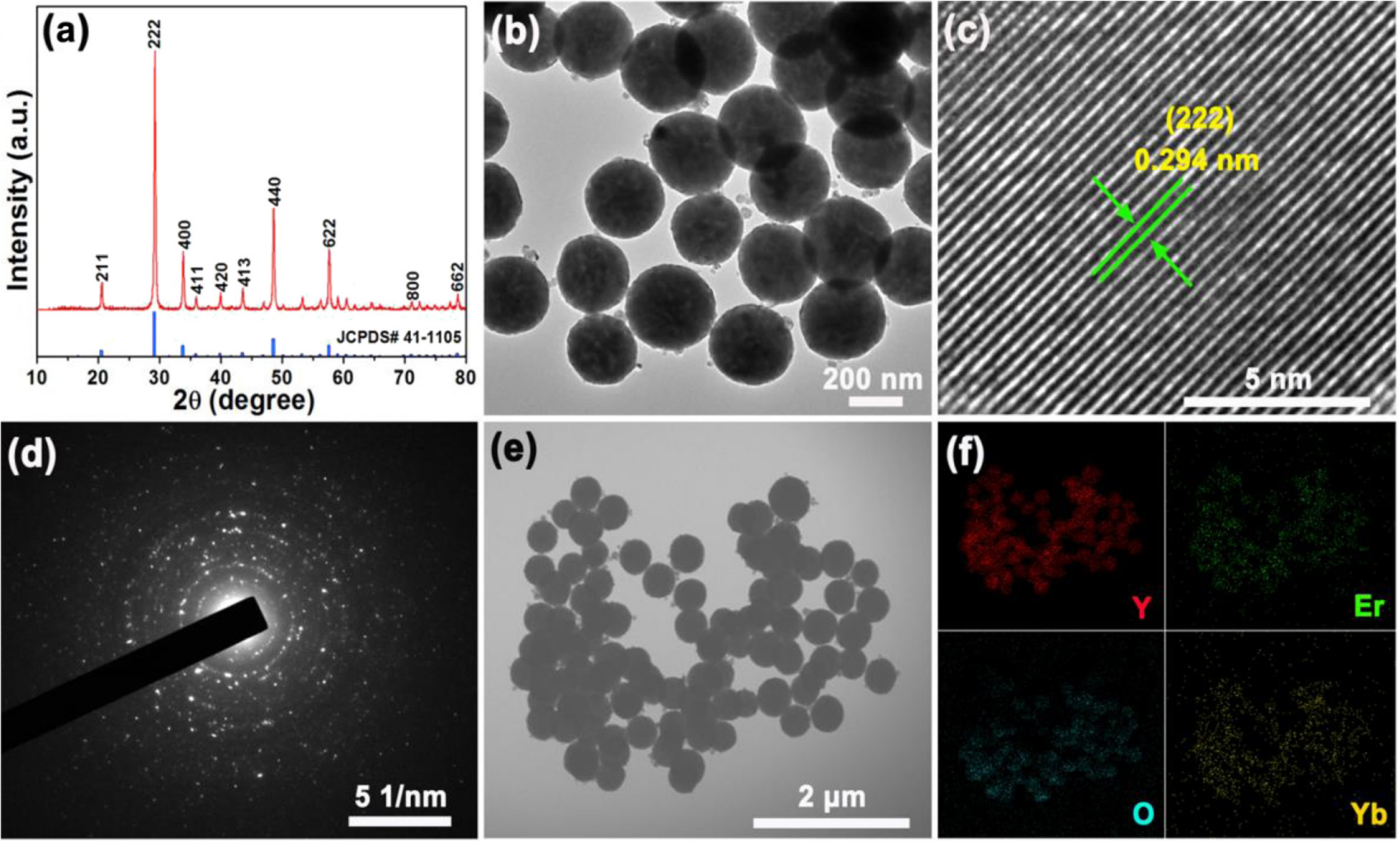
**a** XRD (red solid line) pattern. **b** TEM image. **c** HR-TEM image. **d** SAED pattern. **e** STEM image of Y_2_O_3_:Er^3+^/Yb^3+^ nanoparticles. **f** elemental mapping of Y, O, Er, and Yb

From the XRD pattern in Fig. 1a, it was observed that the diffraction peaks were consistent with the standard pattern (JCPDS#41-1105) and no impurity phases were detected. This means that the powders with a pure cubic phase of Y_2_O_3_ were synthesized and the RE (Er^3+^, Yb^3+^) ions were well diffused into the Y_2_O_3_ host lattice. In Fig. 1b, the TEM image confirmed that the as-prepared Y_2_O_3_:Er^3+^/Yb^3+^ nanoparticles have sphere-like morphologies with the size range of 250–300 nm which is larger than those of the sample prepared by a coprecipitation method (70 nm) [23]. Furthermore, from the high-resolution TEM (HR-TEM) image in Fig. 1c, the interplanar distance between the adjacent lattice planes was estimated to be about 0.294 nm, which is well matched with the (222) plane of the Y_2_O_3_. In Fig. 1d, the selected area electron diffraction (SAED) pattern displayed bright dots and rings. This indicates that the samples are relatively well crystallized. As shown in Fig. 1f, the elemental mapping image taken from the area in the scanning transmission electron microscope (STEM) image of Fig. 1e demonstrates that the constituent materials of Y, O, Er, and Yb are uniformly distributed.

\

**Fig. 2:**
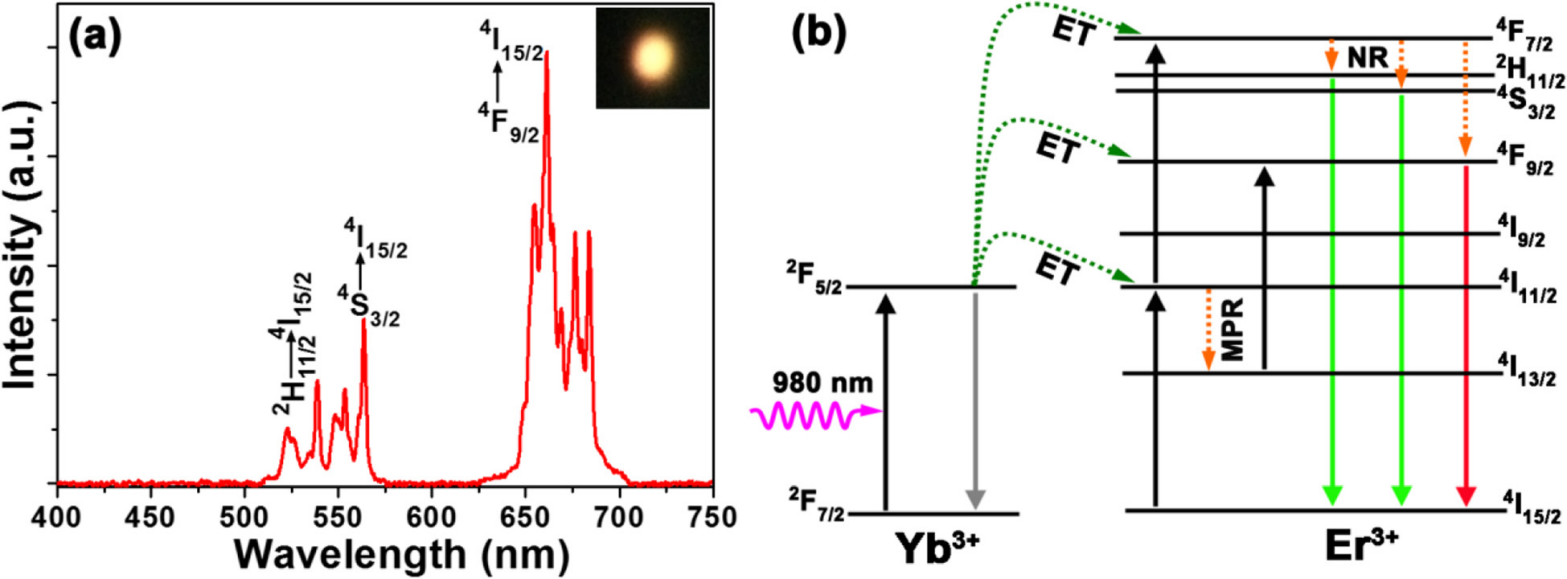
**a** UC spectrum of Y_2_O_3_:Er^3+^/Yb^3+^ nanoparticles (red solid line) under 980 nm light excitation and **b** schematic energy level diagram of Yb^3+^ and Er^3+^ ions and proposed UC mechanism in Y_2_O_3_:Er^3+^/Yb^3+^ nanoparticles under 980 nm light excitation. The inset of **a** shows the photograph taken in the darkness by using a digital camera

Figure 2a, depicts the UC emission spectrum of the Y_2_O_3_:Er^3+^/Yb^3+^ nanoparticles. Under the light excitation at *λ*_ex_ = 980 nm, the fabricated samples exhibited a strong yellow UC emission that can be seen by the naked eye as shown in the inset of Fig. 2a. As shown in Fig. 2a, it is clear that the UC spectrum consists of three distinct bands, that is, two green emissions at *λ* = 510–542 nm and 542–570 nm and strong red emission at *λ* = 625–700 nm. The former is attributed to the ^2^H_11/2_ → ^4^I_15/2_ and ^4^S_3/2_ → ^4^I_15/2_ transitions, respectively and the latter is ascribed to the ^4^ F_9/2_ → ^4^I_15/2_ transition [19, 24]. These results give a reasonable agreement with the previous reports on Er^3+^-doped materials [22, 25]. Here, it is noteworthy that these emission bands are located in the absorption wavelength range of the N-719 dye (i.e., *λ* = 300–800 nm). Thus, the use of Y_2_O_3_:Er^3+^/Yb^3+^ nanoparticles in N-719 dye-based photovoltaic devices would increase the photocurrents due to the enhanced light absorption, except for the sunlight, caused by their green and red light emissions converted from the lights at NIR wavelengths, and thus, the improved device performance of DSSCs could be achieved.

The energy level diagram of Er^3+^ and Yb^3+^ ions including possible UC processes is illustrated in Fig. 2b. Under the light excitation at *λ*_ex_ = 980 nm, the Yb^3+^ ions are excited from the ground sate to the ^2^F_5/2_ level and they drop back. Thus, the energy is transferred to the adjacent Er^3+^ ions, resulting in the population of ^4^I_11/2_ level. Then, the multiphonon relaxation (MPR) process occurs, and part of the ^4^I_11/2_ level decays to the ^4^I_13/2_ level. Meanwhile, the Yb^3+^ ions absorb the second photon energy, and again, the energy is transferred to the adjacent Er^3+^ ions, and the ^4^F_9/2_ and ^4^F_7/2_ levels are populated. Subsequently, the electrons relax to the ^2^H_11/2_, ^4^S_3/2_, and ^4^F_9/2_ levels due to the non-radiative (NR) process. As a result, the strong green and red UC emissions are observed due to the ^2^H_11/2_ → ^4^I_15/2_, ^4^S_3/2_ → ^4^I_15/2_ and ^4^F_9/2_ → ^4^I_15/2_ transitions, respectively.

**Fig. 3:**
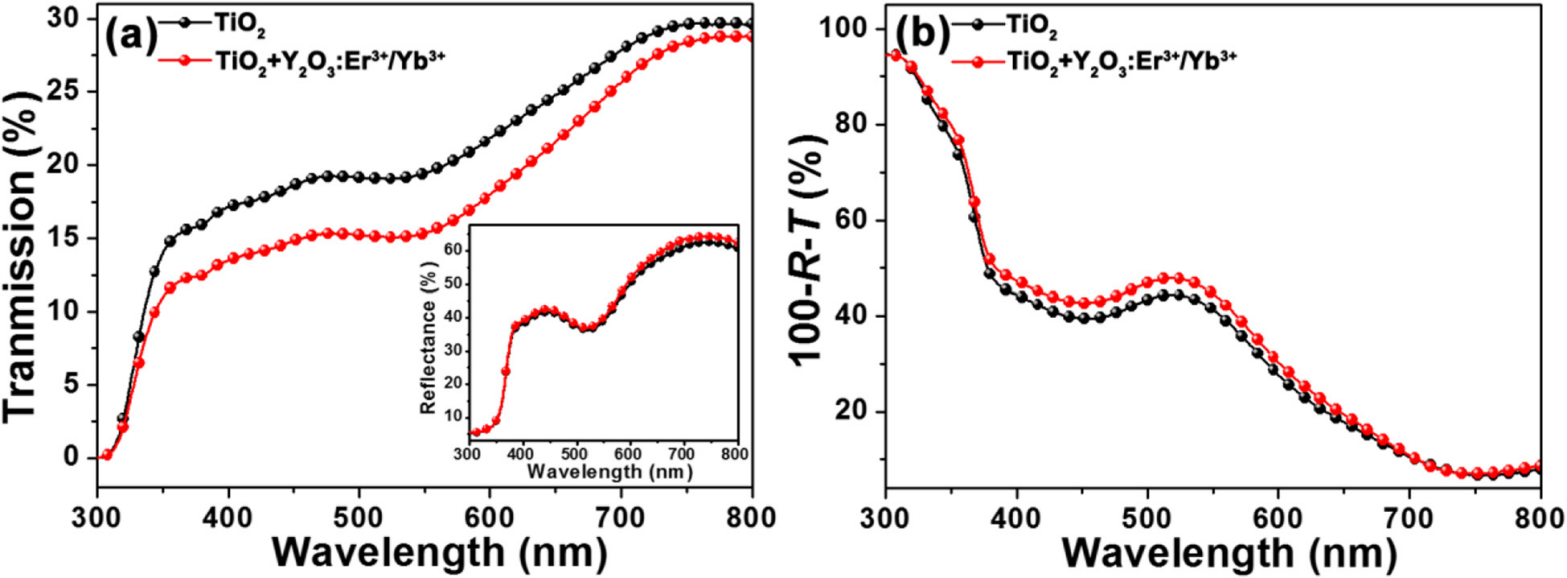
**a** Transmission spectra and **b** estimated absorption spectra of the dye-sensitized TiO_2_ (black solid line) and TiO2 + Y_2_O_3_:Er^3+^/Yb^3+^ (red solid line) photoelectrodes. The inset of **a** shows the reflectance spectra of the corresponding samples

To investigate the light-scattering behaviors of the Y_2_O_3_:Er^3+^/Yb^3+^ nanoparticles in DSSCs, the absorption (i.e., 1-*R*-*T*) of the dye-sensitized photoanodes with the Y_2_O_3_:Er^3+^/Yb^3+^ nanoparticles was extracted by the measured total reflection (*R*) and transmission (*T*) spectra in Fig. 3. As can be seen in Fig. 3a, there were differences in both the total transmission and reflection between the dye-sensitized photoanodes consisting of the TiO_2_ and the TiO_2_ + Y_2_O_3_:Er^3+^/Yb^3+^. Especially, the transmission was further decreased. From these results, as shown in the absorption spectra of Fig. 3b, it can be observed that the dye-sensitized photoanode consisting of the TiO_2_ + Y_2_O_3_:Er^3+^/Yb^3+^ exhibited the relatively higher absorption spectrum compared to one consisting of only TiO_2_ over a wide wavelength region of 350–750 nm. This indicates the enhancement of the light-scattering property in the dye-sensitized photoelectrodes by incorporating the Y_2_O_3_:Er^3+^/Yb^3+^ nanoparticles into the TiO_2_ photoelectrode, and thus, it can lead to the higher photocurrents in DSSCs due to the enhancement in light absorption.

**Fig. 4:**
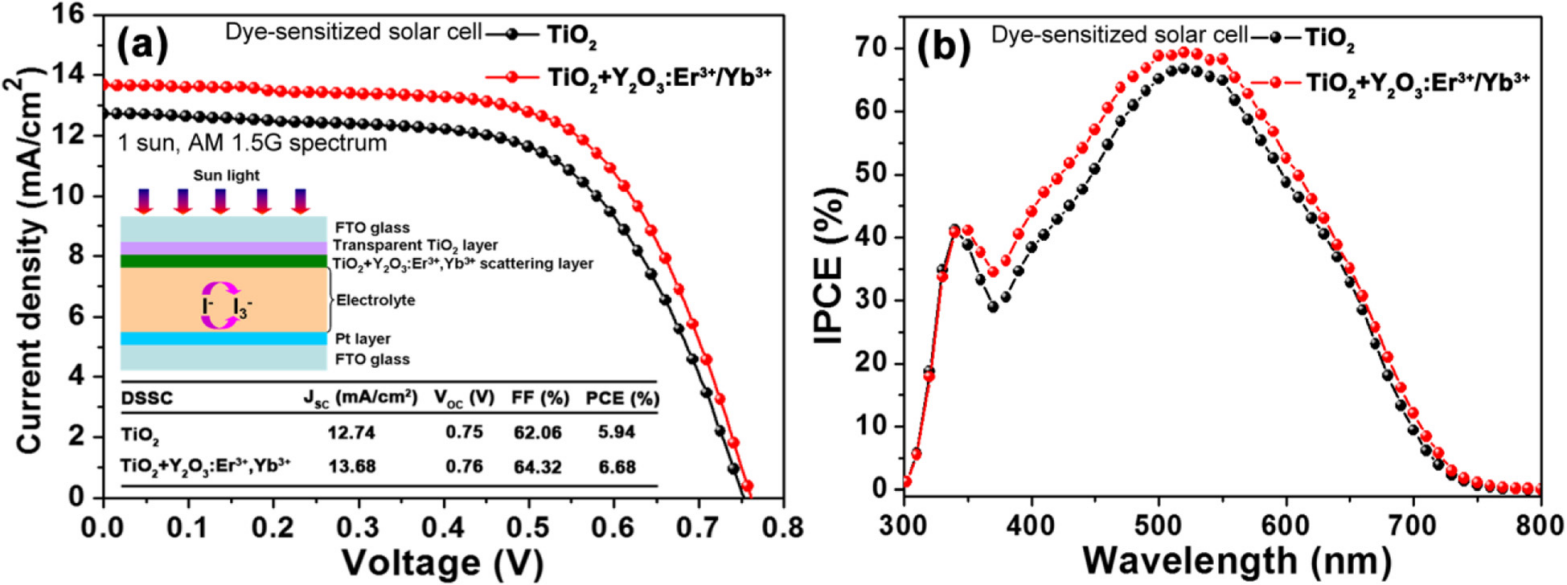
**a** *J*-*V* curves and **b** IPCE spectra of the DSSCs with (red solid line) and without (black solid line) Y_2_O_3_:Er^3+^/Yb^3+^ nanoparticles (1 wt%). The inset of **a** shows the schematic diagram of the DSSC with Y_2_O_3_:Er^3+^/Yb^3+^ nanoparticles and its photovoltaic performance parameters

To investigate the influence of the UC emission phosphors on the photovoltaic performance of DSSCs, the Y_2_O_3_:Er^3+^/Yb^3+^ nanoparticles were introduced into the TiO_2_ photoelectrode. Figure 4 shows (a) the *J*-*V* curves and (b) the *IPCE* spectra of the DSSCs with and without the Y_2_O_3_:Er^3+^/Yb^3+^ nanoparticles. The device characteristics (i.e., short-circuit current density; *J*_SC_, open-circuit voltage; *V*_OC_, fill factor; *FF*, *PCE*) of the corresponding DSSC devices are summarized in the inset of Fig. 4a. The corresponding schematic diagram of the DSSC with the Y_2_O_3_:Er^3+^/Yb^3+^ nanoparticles is also shown. As shown in Fig. 4a, the DSSC without the Y_2_O_3_:Er^3+^/Yb^3+^ nanoparticles exhibited the photovoltaic characteristics of *J*_SC_ = 12.74 mA/cm^2^, *V*_OC_ = 0.75 V, *FF* = 62.06 % and *PCE* = 5.94 %. In contrast, by employing the Y_2_O_3_:Er^3+^/Yb^3+^ nanoparticles in the DSSC, for all the photovoltaic characteristics, the increased values of *J*_SC_ = 13.68 mA/cm^2^, *V*_OC_ = 0.76 V, FF = 64.32 %, and *PCE* = 6.68 % were measured, which leads to the significant *PCE* increment ratio of ~12.4 %. This is mainly because a considerable enhancement (i.e., increment ratio of ~7.4 %) in the *J*_SC_ is caused by the efficient UC emissions of Er^3+^ ions from the NIR light to the visible light as well as the enhanced light-scattering properties [26, 27]. The *V*_OC_ also increased slightly. According to previous reports [27, 28], the *V*_OC_ corresponds to the energy difference between the electronic Fermi level of TiO_2_ film and the redox potential of I^−^/I_3_^−^, i.e., *V*_OC_ = *E*_F(TiO2)_ − *E*_redox_. It is well known that when RE ions are doped and substituted into the Ti^4+^ sites in the TiO_2_, a p-type doping effect appears, resulting in the increment of the Fermi level of the TiO_2_ [29]. Thus, the *V*_OC_ was enhanced. Using the Y_2_O_3_:Er^3+^/Yb^3+^ nanoparticles, the increased photocurrents by the light-scattering effect can be also verified by the *IPCE* data. As can be seen in Fig. 4b, the DSSC with the Y_2_O_3_:Er^3+^/Yb^3+^ nanoparticles showed a higher IPCE spectrum compared to the DSSC without Y_2_O_3_:Er^3+^/Yb^3+^ nanoparticles over an entire spectra range of 350–750 nm. This is well matched with the aforementioned absorption properties in Fig. 3b.

## Conclusions

In summary, the upconverting Y_2_O_3_:Er^3+^/Yb^3+^ phosphor nanoparticles were synthesized and introduced into the TiO_2_ photoelectrode of DSSCs. Under the excitation of the NIR (*λ*_ex_ = 980 nm) light, the strong green and red UC emissions, corresponding to the (^2^H_11/2_, ^4^S_3/2_) → ^4^I_15/2_ and ^4^F_9/2_ → ^4^I_15/2_ transitions, respectively, were observed with the light-scattering effect over a wide wavelength range of 350–750 nm. For the DSCCs incorporated with the Y_2_O_3_:Er^3+^/Yb^3+^ nanoparticles, the enhanced photovoltaic performance was achieved, indicating the increase in the *PCE* value from 5.94 to 6.68 % (i.e., *PCE* increment ratio of ~12.4 %). These results can provide a better insight into the phosphor nanoparticles with the NIR sunlight-upconverting functions into the visible lights as well as the light-scattering effect for high-performance dye-sensitized photovoltaic devices.
